# Strong Association of the Myriad Discrete Speckled Nuclear Pattern With Anti-SS-A/Ro60 Antibodies: Consensus Experience of Four International Expert Centers

**DOI:** 10.3389/fimmu.2021.730102

**Published:** 2021-10-05

**Authors:** Nadja Röber, Alessandra Dellavance, Fernanda Ingénito, Marie-Luise Reimer, Orlando Gabriel Carballo, Karsten Conrad, Edward K. L. Chan, Luis E. C. Andrade

**Affiliations:** ^1^Institute of Immunology, Technical University Dresden, Dresden, Germany; ^2^Division of Research and Development, Fleury Medicine and Health Laboratories, São Paulo, Brazil; ^3^Immunology Laboratory, Durand Hospital, Buenos Aires, Argentina; ^4^Department of Oral Biology, University of Florida, Gainesville, FL, United States; ^5^Division of Immunology, Fleury Medicine and Health Laboratories, São Paulo, Brazil; ^6^Division of Rheumatology, Universidade Federal de São Paulo, São Paulo, Brazil

**Keywords:** autoantibodies, antinuclear antibodies, SS-A/Ro antibodies, autoimmune disease, immunofluorescence patterns, HEp-2 cells, ICAP, HEp-2 IFA

## Abstract

**Introduction:**

The morphological patterns in indirect immunofluorescence assay on HEp-2 cells (HEp-2 IFA) reflect the autoantibodies in the sample. The International Consensus on ANA Patterns (ICAP) classifies 30 relevant patterns (AC-0 to AC-29). AC-4 (fine speckled nuclear pattern) is associated to anti-SS-A/Ro, anti-SS-B/La, and several autoantibodies. Anti-SS-A/Ro samples may contain antibodies to Ro60 and Ro52. A variation of AC-4 (herein designated AC-4a), characterized by myriad discrete nuclear speckles, was reported to be associated with anti-SS-A/Ro. The plain fine speckled pattern (herein designated AC-4b) seldom was associated with anti-SS-A/Ro. This study reports the experience of four expert laboratories on AC-4a and AC-4b.

**Methods:**

Anti-Ro60 monoclonal antibody A7 was used to investigate the HEp-2 IFA pattern. Records containing concomitant HEp-2 IFA and SS-A/Ro tests from Durand Laboratory, Argentina (*n* = 383) and Fleury Laboratory, Brazil (*n* = 144,471) were analyzed for associations between HEp-2 IFA patterns and disease-associated autoantibodies (DAA): double-stranded DNA, Scl-70, nucleosome, SS-B/La, Sm, and U1-RNP. A total of 381 samples from Dresden Technical University (TU-Dresden), Germany, were assayed for HEp-2 IFA and DAA.

**Results:**

Monoclonal A7 recognized Ro60 in Western blot and immunoprecipitation, and yielded the AC-4a pattern on HEp-2 IFA. Analyses from Durand Laboratory and Fleury Laboratory yielded compatible results: AC-4a was less frequent (8.9% and 2.7%, respectively) than AC-4b (26.1% and 24.2%) in HEp-2 IFA-positive samples. Reactivity to SS-A/Ro occurred in 67.6% and 96.3% of AC-4a-pattern samples against 23% and 6.8% of AC-4b pattern samples. Reciprocally, AC-4a occurred in 24% and 47.1% of anti-SS-A/Ro-positive samples, and in 3.8% and 0.1% of anti-SS-A/Ro-negative samples. Data from TU-Dresden show that the AC-4a pattern occurred in 69% of 169 anti-SS-A/Ro-monospecific samples (62% of all anti-SS-A/Ro-positive samples) and in 4% of anti-SS-A/Ro-negative samples, whereas anti-SS-A/Ro occurred in 98.3% of AC-4a samples and in 47.9% of AC-4b samples. In all laboratories, coexistence of anti-SS-B/La, but not other DAA, in anti-SS-A/Ro-positive samples did not disturb the AC-4a pattern. AC-4a was predominantly associated with anti-Ro60 antibodies.

**Conclusions:**

This study confirms the association of AC-4a pattern and anti-SS-A/Ro in opposition to the AC-4b pattern. The results of four international expert laboratories support the worldwide applicability of these AC-4 pattern variants and their incorporation into ICAP classification under codes AC-4a and AC-4b, respectively. The AC-4 pattern should be maintained as an umbrella pattern for cases in which one cannot discriminate AC-4a and AC-4b patterns. The acknowledgment of the AC-4a pattern should add value to HEp-2 IFA interpretation.

## Introduction

The indirect immunofluorescence assay on HEp-2 cells (HEp-2 IFA), traditionally known as the antinuclear antibody (ANA) test, is widely used as an initial approach for the screening for autoantibodies in systemic rheumatic autoimmune diseases (SARD) (1, 2). IFA using a monolayer of HEp-2 cells allows the identification of several morphological patterns that mirror the topographic distribution of autoantigens recognized by autoantibodies in a given sample. Thus, HEp-2 IFA patterns indicate the putative autoantibody specificities in the sample and represent a valuable parameter for the interpretation of a positive HEp-2 IFA test (3, 4). For example, the homogeneous nuclear pattern (associated with antibodies to native DNA and nucleosome) and the coarse speckled nuclear pattern (associated with antibodies to Sm and U1RNP) deserve serious attention and further investigation with reflex autoantibody testing (3, 4). In contrast, the dense fine speckled nuclear pattern (strongly associated with anti-DFS70 antibodies) is most probably not related to systemic autoimmune diseases, even at high titer (4, 5). The acknowledged clinical utility of the HEp-2 IFA patterns stimulated specialists to set up the International Consensus on ANA Patterns initiative (ICAP) (6). ICAP has established a classification algorithm comprising 30 relevant HEp-2 IFA patterns with the respective alphanumeric codes from AC-0 (AC, for anti-cell) to AC-29 (www.anapatterns.org).

Some AC patterns have strong and circumscribed immunologic and clinical associations (7). For example, AC-1 (homogeneous nuclear pattern) and AC-5 (coarse speckled nuclear pattern) are strongly associated with anti-native DNA and/or nucleosome antibodies, which are valuable biomarkers for systemic lupus erythematosus (SLE) (8, 9). The composite AC-29 pattern is tightly associated with antibodies to DNA topoisomerase 1 (10–12), which is a biomarker for systemic sclerosis. On the other hand, some AC patterns are not associated with systemic autoimmune diseases. The prototype pattern in this category is the dense fine speckled nuclear pattern (AC-2) that preferentially is observed in asymptomatic individuals (4, 5, 13) and in patients with non-autoimmune diseases (14). Finally, some AC patterns have heterogeneous clinical relevance (7). In the latter category, the AC-4 (nuclear fine speckled) pattern deserves attention because this is one of the most frequent patterns observed in the clinical laboratory routine. Mariz et al. reported that the AC-4 pattern corresponds to 45.8% and 42% of all HEp-2 IFA-positive samples from normal individuals and patients with SARD, respectively (4). Although the AC-4 pattern has been associated with antibodies to SS-A/Ro and/or SS-B/La (3, 7), it has much higher frequency in the general population and in SARD patients than the expected frequency of these two autoantibodies. This observation defies the traditional association of the AC-4 pattern with anti-SS-A/Ro and anti-SS-B/La, suggesting the possibility that subtle nuances may help discriminate the HEp-2 IFA pattern truly associated with these antibodies.

In fact, Dellavance et al. identified a variation of the AC-4 pattern that was strongly associated with antibodies to the anti-Ro60 moiety of the SS-A/Ro antigen system (15). Originally described as myriad tiny discrete speckles across the nucleoplasm of interphase cells and not staining the mitotic chromosome masses, this AC-4 variant pattern was present in 91.6% of 48 sequential samples positive for anti-Ro60 antibodies. Conversely, anti-Ro60 reactivity was demonstrated in 98.8% of 86 consecutive samples presenting the AC-4 variant pattern. These investigators proposed that the AC-4 variant pattern should prompt the reflex testing for anti-Ro60 antibodies.

**Figure 1:**
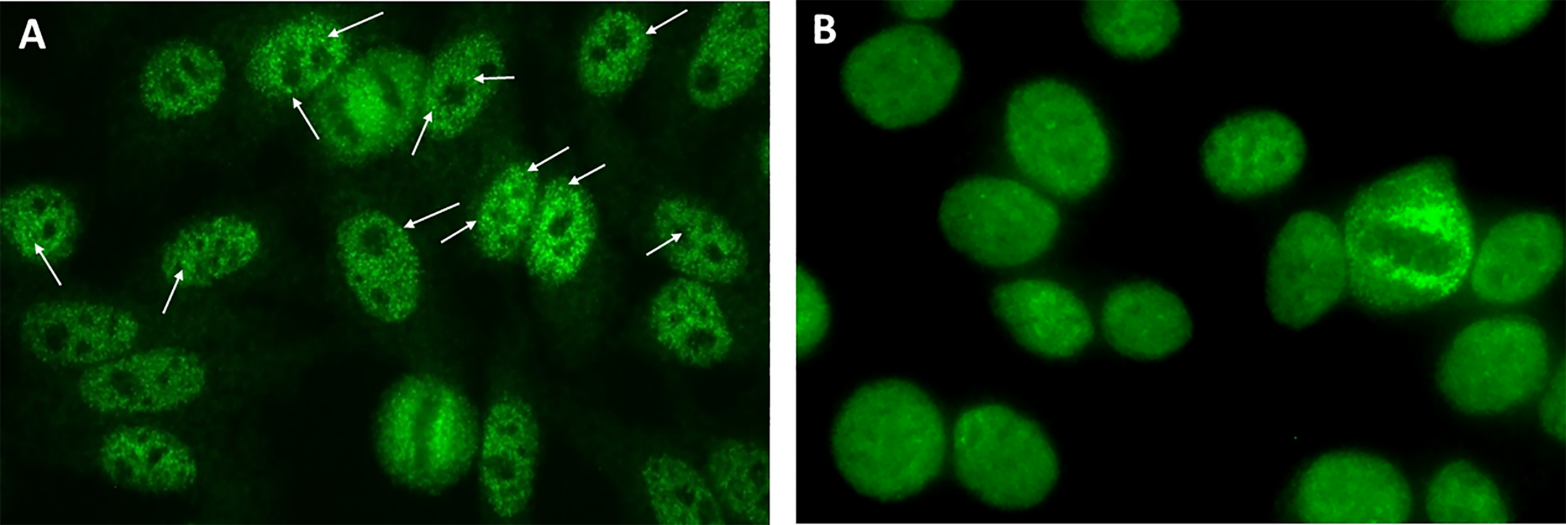
Indirect immunofluorescence on HEp-2 cells showing the AC-4a and AC-4b patterns. **(A)** IUIS/ASC reference human serum for anti-SS-A/Ro IS2105 diluted 1/160 exhibiting the characteristic myriad discrete speckled nuclear AC-4a pattern (arrows, discrete tiny nuclear speckles); **(B)** Human serum with no reactivity to SS-A/Ro, diluted 1/160, exhibiting the characteristic plain nuclear fine speckled pattern (AC-4b) mostly lacking discrete speckles. Inova HEp-2 slide used. Magnification ×400.

The present study reports the experience of four independent expert laboratories in Argentina, Brazil, Germany, and the USA, respectively, regarding the autoantibody associations of the traditional AC-4 and the variant AC-4 patterns. In this study, the myriad tiny discrete speckles AC-4 variant pattern will be preliminarily designated AC-4a and the plain AC-4 pattern will be designated AC-4b (**Figure 1**). The term anti-SS-A/Ro will be used to describe reactivity to Ro60 and/or Ro52, whereas the terms anti-Ro60 and anti-Ro52 will be used when only one of these autoantibodies is present.

## Materials and Methods

**Figure 2:**
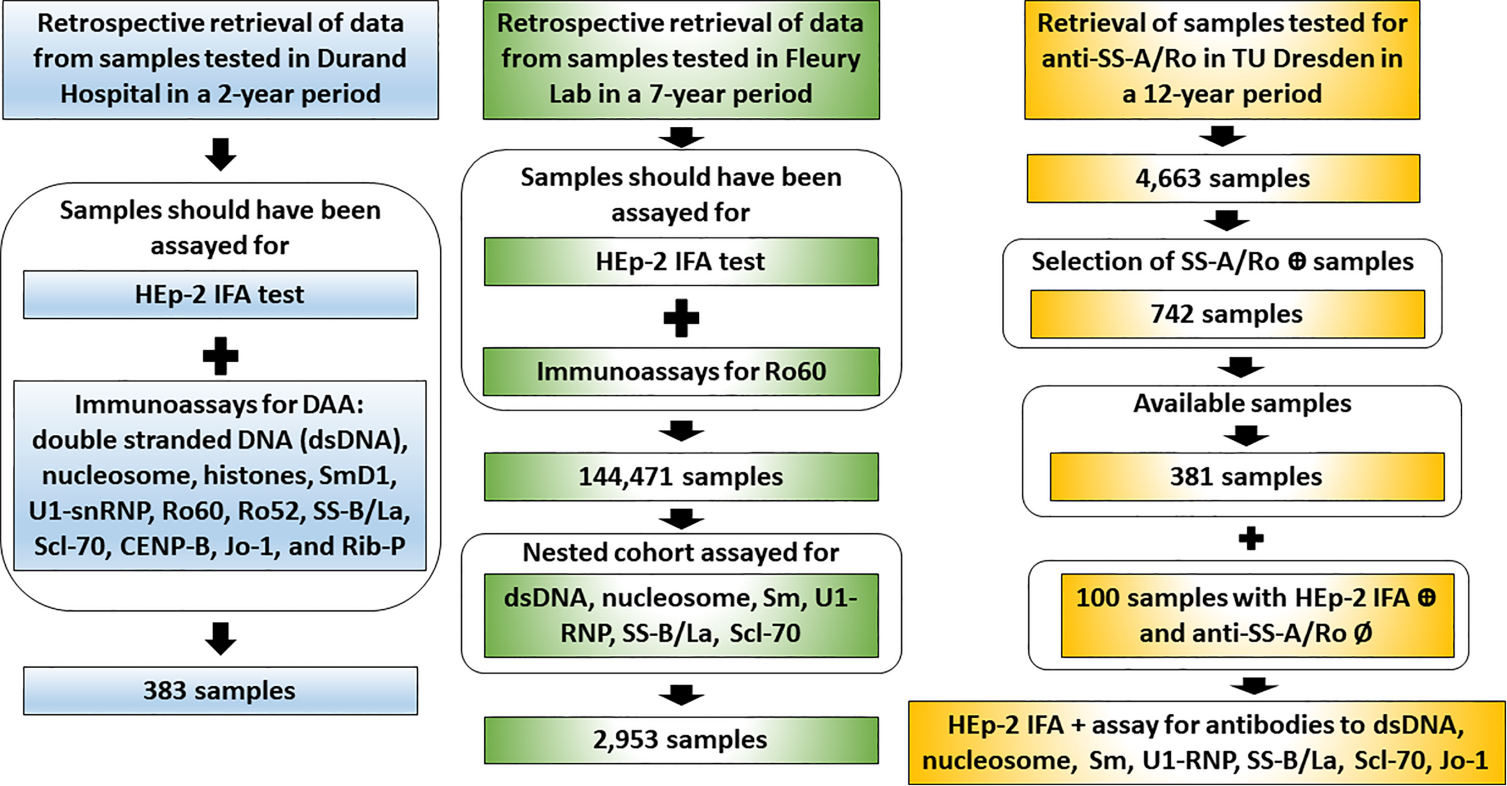
Workflow of data retrieval and sample analysis in the three independent clinical laboratories. The Argentinian center (Durand Hospital) and the Brazilian center (Fleury Laboratory) provided retrospective data obtained from databanks of samples assayed in the day-to-day operation. From the serum bank, the German center (Technical University Dresden) retrieved samples recorded as positive or negative for anti-SS-A/Ro and prospectively assayed them for disease-associated autoantibodies (DAA).

This study is based on the analysis of clinical laboratory databanks and experimental data from four independent international laboratories run by autoantibody experts belonging to the ICAP executive board. Therefore, the methodological approach is slightly different for each center (**Figure 2**). For the Argentinian and Brazilian centers, data on immunoassay results were retrieved from established databanks and no additional sample processing was done. These two centers specify the two AC-4 variants in their routine operation for several years. For the German center, samples were retrieved from a serum bank and re-processed specially for this study. The USA center contributed the analysis of monoclonal antibody and an international standard reference serum with reactivity to Ro60. In all centers, HEp-2 IFA patterns were defined according to the ICAP classification, with the additional classification of the AC-4a and AC-4b patterns (**Figures 1A, B**). The chi-square test was used for comparison of the distribution of frequency of two or more categorical variables and a *p*-value <0.05 was considered statistically significant.

### Monoclonal Antibody Against SS-A/Ro 60-kDa Protein

A human Ro60 full-length cDNA (16) was subcloned into pET28 expression vector (Invitrogen, Carlsbad, CA, USA). Recombinant proteins were produced in *Escherichia coli* BL21 (DE3; Novagen, Madison, WI, USA) and purified using Ni^2+^ affinity chromatography as per the manufacturer’s instructions (Qiagen, Valencia, CA, USA) (17). The soluble recombinant protein was subsequently used in the immunization protocol and production of murine hybridomas using standard protocols as previously described (18, 19). Monoclonal anti-Ro60 IgG antibody A7 was detected in initial screening by ELISA and followed by using HEp-2 IFA. Further characterization of A7 included Western blot analysis using soluble lysate from MOLT-4 cells and a higher ratio of acrylamide monomer as cross-linker in an optimized polyacrylamide gel electrophoresis system (20), immunoprecipitation (IP) with lysate from HeLa cells metabolically labeled with [^35^S]-methionine (21, 22), and also IP using *in vitro* [^35^S]-methionine-labeled translation products of Ro60 recombinant cDNA (19). IP was performed in the presence of unconjugated goat anti-mouse IgG/IgM affinity-purified IgG (Jackson ImmunoResearch Lab, West Grove, PA, USA) as a secondary linker to ensure binding of the putative monoclonal antibody to the protein G-Sepharose beads.

### Argentinian Branch

The study included 383 samples consecutively submitted for HEp-2 IFA testing and disease-associated autoantibodies (DAA) for a 2-year period (2017 and 2018) at the Immunology Laboratory of Durand Hospital in Buenos Aires, Argentina. HEp-2 IFA was performed using slides from Bio-Rad Kallestad™ (Hercules, CA, USA) according to the manufacturer’s instructions and observed under ×400 magnification by two expert technicians. The selected samples were tested for DAA, including double-stranded DNA (dsDNA), nucleosome, histones, SmD1, U1-snRNP, Ro60, Ro52, SS-B/La, Scl-70, CENP-B, Jo-1, and Rib-P, using IgG Line Immuno Assay (LIA) kits (IMTEC-ANA-LIA) from HUMAN (Wiesbaden, Germany). The use of data from the databank was approved by the Ethics Committee at Durand Hospital.

### Brazilian Branch

The retrospective analysis comprehended results obtained in 144,471 serum samples tested for HEp-2 IFA and anti-Ro60 antibodies in the period from January 2012 to July 2018. A nested cohort of 2,953 samples had concomitant results for tests for other DAA (dsDNA, nucleosome, Sm, U1-RNP, SS-B/La, Scl-70). HEp-2 IFA was performed using HEp-2 slides from MBL-Bion Enterprise Ltd (Des Plaines, IL, USA), according to the manufacturer’s instructions at ×400 magnification by at least two expert observers in a team of 12 analysts intensively trained and harmonized to interpret HEp-2 IFA. Determination of antibodies to Ro60, SS-B/La, Sm, U1-RNP, Jo-1, and Scl-70 was performed by the Ouchterlony double immunodiffusion (DID) technique (23). It is relevant to mention that this method is able to detect antibodies to Ro60 but largely insensitive for antibodies to Ro52 (24). Therefore, the data from the Brazilian branch will be referred to as anti-Ro60 antibodies. Antibodies to ds-DNA and to nucleosome were determined by IFA on *Crithidia luciliae* (25) and chemoluminescence immunoassay (Inova Diagnostics, La Jolla, CA, USA), respectively. The use of data from the databank was approved by the Ethics Committee at Fleury Laboratory.

### German Branch

**Figure 3:**
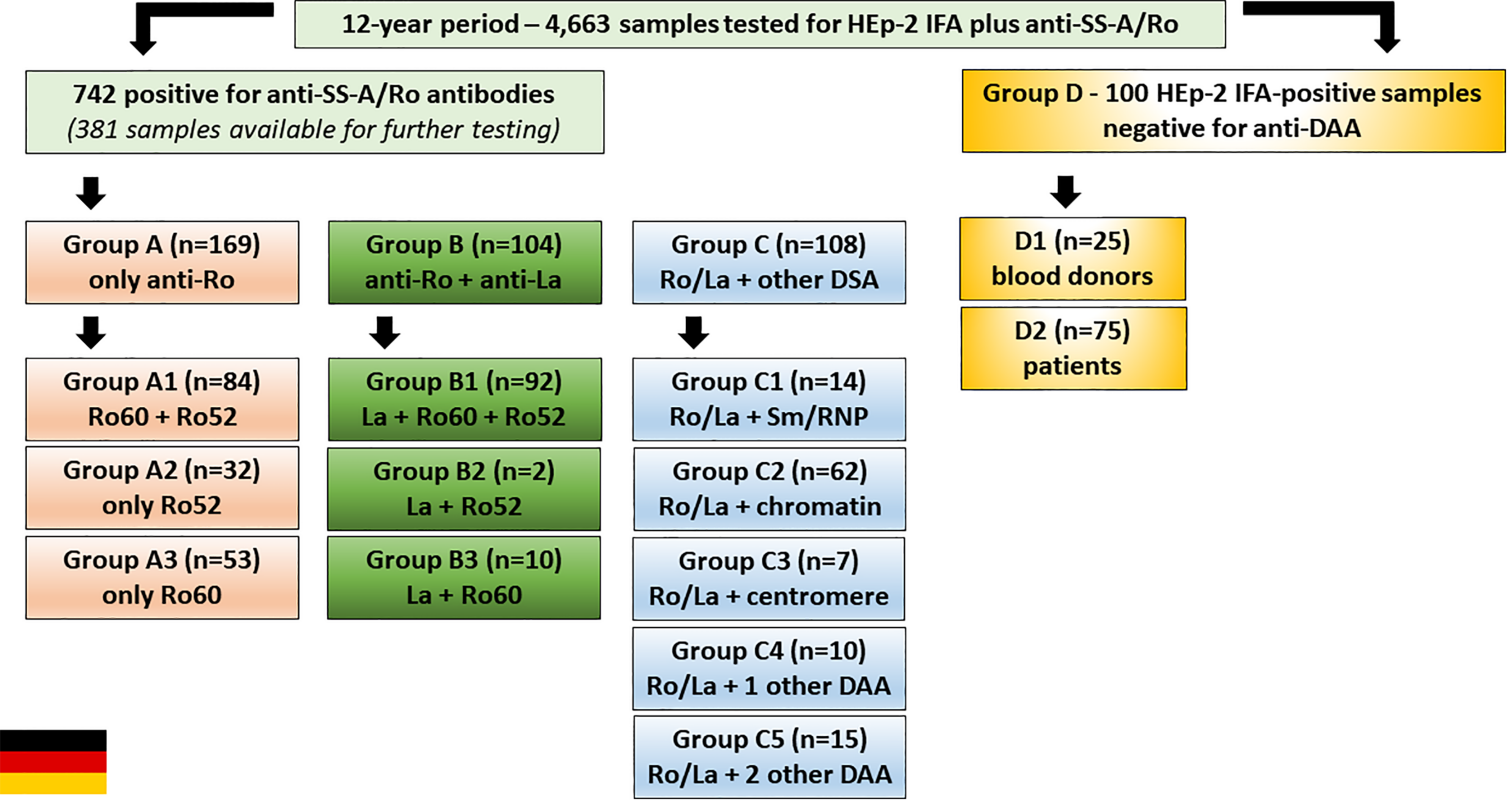
Serum samples from the Institute of Immunology of the Technical University of Dresden, Germany, were classified into four groups. Group A: samples with antibodies to SS-A/Ro and no other disease-associated autoantibody (DAA) (dsDNA, nucleosome, Sm, U1-RNP, SS-B/La, Scl-70, Jo-1). Group B: samples with antibodies to SS-A/Ro and SS-B/La, and no additional DAA. Group C: samples with anti-SS-A/Ro antibodies (with or without anti-SS-B/La) and one or more additional DAA. Group D: samples with positive HEp-2 IFA result and negative for anti-SS-A/Ro and other DAA. Each group was further stratified as shown in the boxes.

Samples were retrieved from a 12-year period serum bank at the Institute of Immunology of the Technical University Dresden (TU Dresden). From 2001 to 2012, 4,663 samples have been tested for HEp-2 IFA using slides from Medipan GmbH (Dahlewitz, Germany) and for anti-SS-A/Ro using ELISA made by Orgentec Diagnostica GmbH (Mainz, Germany). Out of these, 742 samples were reagent against SS-A/Ro (Ro60 and/or Ro52), of which 381 were available for further testing including separate ELISA for antibodies to Ro60 and Ro52 made by Orgentec Diagnostica GmbH, Mainz, Germany). As a control, 100 samples were randomly chosen from HEp-2 IFA-positive samples and tested non-reagent to SS-A/Ro and other DAA (dsDNA, nucleosome, Sm, U1-RNP, SS-B/La, Scl-70, Jo-1). This control group (HEp-2 IFA-positive, but negative for DAA) was formed by 75 samples from patients and 25 samples from blood donors, all stored in the TU Dresden serum bank (**Figure 3**).

The 381 anti-SS-A/Ro-positive samples from the serum bank were divided into three groups (**Figure 3**): Group A (169 samples with monospecific anti-SS-A/Ro reactivity); Group B (104 samples with reactivity to SS-A/Ro and SS-B/La); Group C [108 samples with reactivity to SS-A/Ro (with or without anti-SS-B/La) plus at least one additional DAA]. Group A was further divided into subgroup A1 (84 samples with reactivity to Ro52 and Ro60), subgroup A2 (32 samples with reactivity to Ro52 and negative for anti-Ro60), and subgroup A3 (53 samples reactive with Ro60 and negative for anti-Ro52). Analogously, Group B was further divided into subgroup B1 (92 samples reactive with SS-B/La, Ro52 and Ro60), subgroup B2 (two samples reactive with SS-B/La and Ro52, but negative for anti-Ro60), and subgroup B3 (10 samples reactive with SS-B/La and Ro60, but negative for anti-Ro52). Group C was further divided into subgroup C1 (14 samples with additional reactivity to Sm and/or U1-RNP), subgroup C2 (62 samples with additional reactivity to chromatin-associated antigens), subgroup C3 (7 samples with additional reactivity to centromere), subgroup C4 (10 samples with reactivity to one additional autoantibody), and subgroup C5 (15 samples with antibodies to at least two additional autoantibodies).

Group D was formed with 100 freshly obtained HEp-2 IFA-positive anti-SS-A/Ro-negative samples from two sources, routine HEp-2 IFA testing (subgroup D1) and blood donors (subgroup D2). Subgroup D1 was randomly chosen among samples with successive HEP-p-2 IFA titer (1/80, 1/160, up to 1/2560) and a negative result for anti- SS-A/Ro. After removing those with a positive result for DAA and after exclusion of follow-up duplicate sera from the same individual, there were 75 SS-A/Ro-negative samples in subgroup D1. Subgroup D2 came from blood donors and comprised 25 samples from anti-SS-A/Ro-negative blood donors that were also negative for other autoantibodies.

The use of serum samples for this study was approved by the Ethics Committee of the Medical Faculty Carl Gustav Carus of the TU Dresden (EK 56022014, EK 226112006) and of the Sächsische Landesärztekammer (EK-BR-13/13-1). HEp-2 IFA was performed using ANA HEp-2 plus slides (Medipan GmbH, Dahlewitz, Germany) according to the manufacturer’s instructions and observed under ×400 magnification by two expert technicians.

## Results

### Monoclonal A7 With Reactivity to Ro60 Reproduces the Characteristic AC-4a Pattern

**Figure 4:**
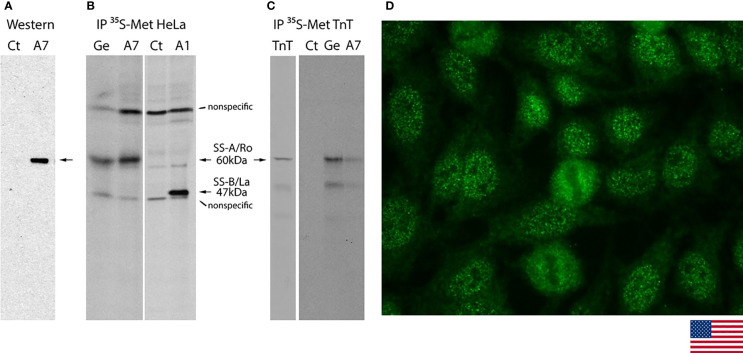
Characterization of SS-A/Ro60 monoclonal antibody A7 in Western blotting, immunoprecipitation, and indirect immunofluorescence on HEp-2 cells. **(A)** Western blot analysis of A7 showed reactivity to the 60 kDa protein (arrow) in the MOLT-4 cell lysate. Control (Ct) culture supernatant showed no reactivity. **(B)** Immunoprecipitation (IP) with lysate from HeLa cells metabolically labeled with [^35^S]-methionine. Ge and A1 are human prototype serum for anti-SS-A/Ro and mouse monoclonal antibody to SS-B/La, respectively. The non-specific bands showing in all lanes were seen when secondary bridging antibody was used. **(C)** IP with *in vitro* transcription and translation (TnT) product of Ro60. The lane TnT represented loading of 1/10 amount of [^35^S]-methionine-labeled product compared to the one used in IP with Ct, Ge, and A7. **(D)** Indirect immunofluorescence on HEp-2 cells with monoclonal A7 against Ro60 showing the characteristic AC-4a pattern (magnification ×400).

Hybridoma A7 was selected as one of the strong reactors in the initial screening with Ro60 ELISA. After two rounds of subcloning, demonstration for monoclonal A7 to be specific for Ro60 was provided by Western blot analysis and IP. A7 recognized a single 60-kDa band in Western blot analysis of MOLT-4 cell lysate (**Figure 4A**). IP with radiolabeled HeLa cell lysate showed reactivity to a 60-kDa protein that co-migrates with that of the prototype anti-SS-A/Ro serum Ge (**Figure 4B**) and not detected with control culture medium (Ct) or monoclonal antibody to SS-B/La A1 (18). A7 also shows reactivity to recombinant Ro60 in IP with labeled *in vitro* translation product (**Figure 4C**). HEp-2 IFA with A7 showed the characteristic AC-4a pattern (**Figure 4D**) similar to the staining with IUIS/ASC reference serum for anti-SS-A/Ro IS2105 (**Figure 1A**).

Thus, the characteristic features of the AC-4a pattern observed with anti-SS-A/Ro-positive human samples were reproduced with the mouse monoclonal A7 with reactivity to the Ro60. Next, we show that the association between anti-SS-A/Ro antibodies and the AC-4a pattern is largely confirmed with serum collections from three independent international expert clinical laboratories.

### Argentinian Branch

**Figure 5:**
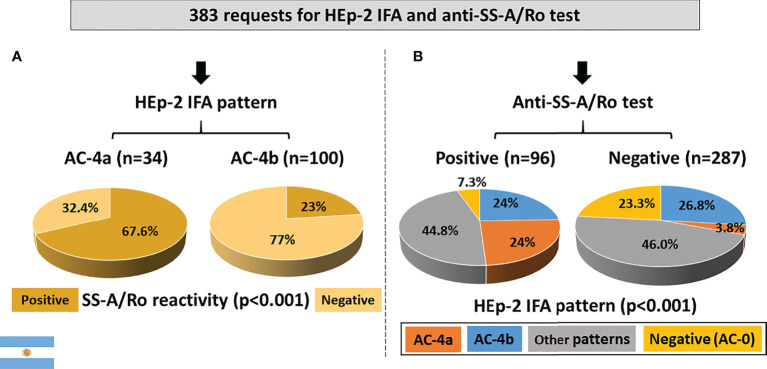
Association between the AC-4a pattern and antibodies to SS-A/Ro according to data from the Immunology Laboratory of Durand Hospital in Buenos Aires, Argentina. **(A)** HEp-2 IFA pattern perspective: most samples with the AC-4a pattern were positive, whereas most samples with the AC-4b were negative for anti-SS-A/Ro. **(B)** Anti-SS-/Ro antibody perspective: the AC-4a pattern was prevalent in samples positive for anti-SS-A/Ro60 and rare in negative samples. In contrast, the AC-4b pattern showed similar frequency in samples positive and negative for anti-SS-A/Ro.

Out of 383 samples with concomitant request for HEp-2 IFA tests and DAA, 309 had a positive HEp-2 IFA result. Among these, the AC-4a pattern was less frequent (34; 8.9%) than the AC-4b pattern (100; 26.1%). Reactivity to SS-A/Ro was observed in the majority (67.6%) of samples with the AC-4a pattern. In contrast, among the 100 samples presenting the AC-4b pattern, less than one-quarter presented antibodies to SS-A/Ro (**Figure 5A**).

From the perspective of reactivity to SS-A/Ro, among the 96 anti-SS-A/Ro-positive samples, 23 (24%) presented the AC-4a pattern, 23 (24%) presented the AC-4b pattern, 43 (44.8%) presented other patterns, and 7 (7.3%) presented a negative HEp-2 IFA result. In contrast, the AC-4a pattern occurred in a minority (3.8%) of the 287 SS-A/Ro-negative samples, which presented preferentially the AC-4b pattern (26.8%), other patterns (46%), and negative result (23.3%) (**Figure 5B**). The difference in AC-4a pattern distribution between samples with and without reactivity to SS-A/Ro was statistically significant.

**Figure 6:**
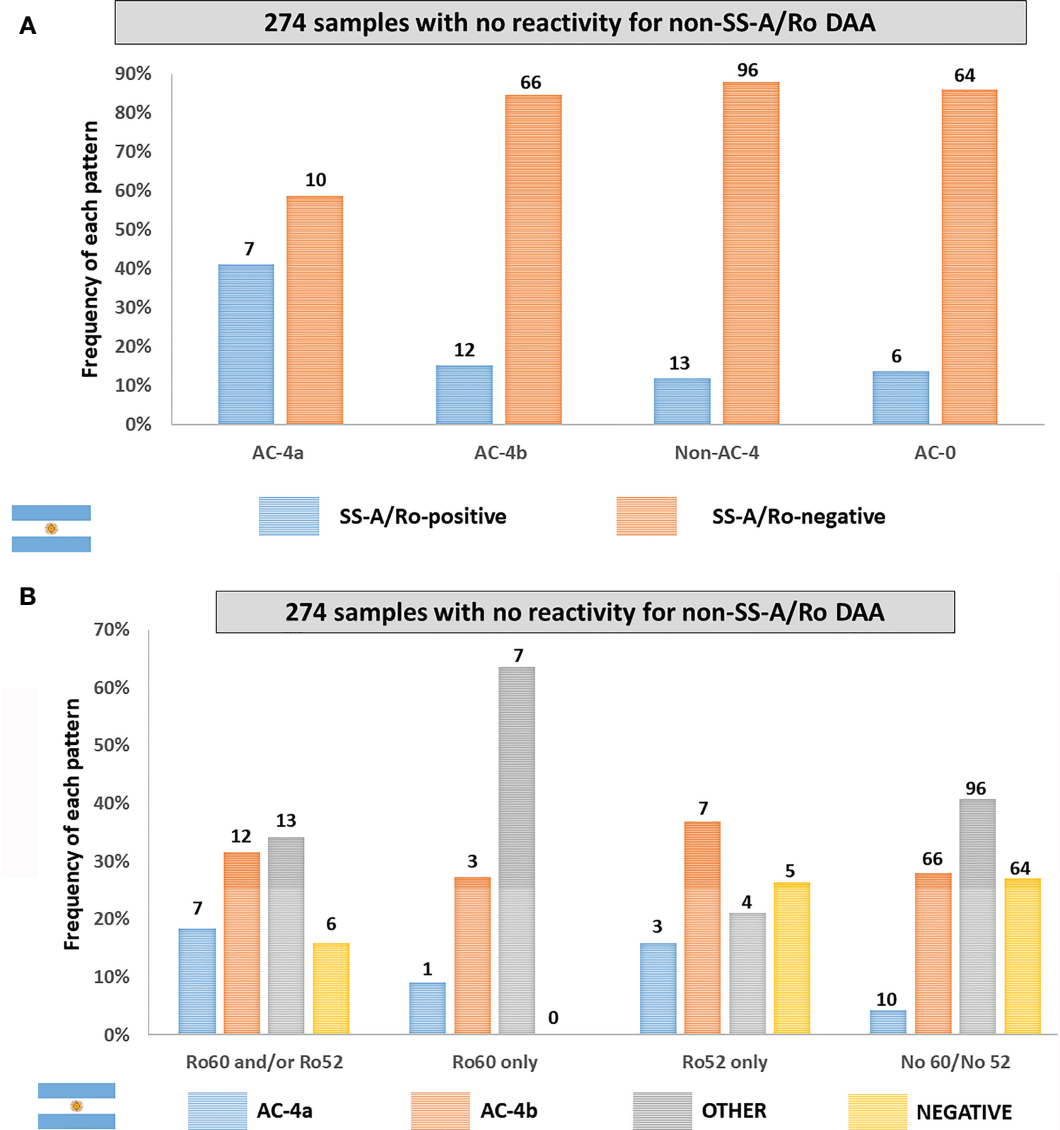
Frequency of the AC-4a pattern and other patterns in samples with no disease-associated autoantibody (DAA) other than anti-SS-A/Ro according to data from the Immunology Laboratory of Durand Hospital in Buenos Aires, Argentina. This analysis included only samples that were negative for non-SS-A/Ro DAA: dsDNA, chromatin, histones, SmD1, U1-RNP, SS-B/La, Scl-70, CENP-B, Jo-1, and Ribosomal-P. **(A)** Samples were classified according to the presence or absence of antibodies to SS-A/Ro. The *y*-axis represents the percentage frequency for each pattern. **(B)** Samples were classified according to the fine specificity of anti-SS-A/Ro antibodies (anti-Ro60 and anti-Ro52). The *y*-axis represents the percentage frequency for each autoantibody combination. Absolute numbers in each category are shown at the top of columns.

Out of the 383 samples, 274 had confirmed negative result for all tested non-SS-A/Ro DAA, including dsDNA, chromatin, histone, SS-B/La, Sm, U1-RNP, Jo-1, Scl-70, CENP-B, and P-ribosomal protein. Reactivity to SS-A/Ro was observed in 7 (41.2%) of the 17 samples showing the AC-4a pattern, but in a minority (15.4%) of the 78 samples with the AC-4b pattern as well as in samples showing non-AC-4 patterns (11.9%) and in non-reagent (AC-0) samples (8.6%) (**Figure 6A**). The AC-4a pattern was not sensitive for the presence of anti-SS-A/Ro antibodies, as among the 38 anti-SS-A/Ro-positive samples, 7 (18.4%) presented the AC-4a pattern, 12 (31.6%) presented the AC-4b pattern, 13 (34.2%) presented non-AC-4 patterns, and 6 (15.8%) presented a negative HEp-2 IFA result. The frequency of the AC-4a pattern increased to 66.7% in the 15 samples with coexistence of anti-SS-A/Ro and anti-SS-B/La antibodies. Of note, there were six samples with exclusive reactivity to SS-B/La, none of which presented the AC-4a or the AC-4b patterns. One was non-reagent in HEp-2 IFA (AC-0), one had the AC-8 pattern, two had the pure AC-1 pattern, and two had the AC-1 pattern combined with AC-19 and AC-8 patterns, respectively. The AC-4a pattern showed high specificity for anti-SS-A/Ro antibodies, as this pattern occurred in only 10 (4.2%) of 236 samples with no reactivity to SS-A/Ro. **Figure 6B** shows the fine specificity of anti-SS-A/Ro antibodies, indicating that the AC-4a pattern was observed in samples with reactivity to Ro60 or to Ro52.

Out of the 383 samples, 109 presented at least one non-SS-A/Ro DAA antibody, 58 of which had reactivity to SS-A/Ro. The AC-4a pattern occurred in 16 (27.6%) of the 58 anti-SS-A/Ro-positive samples, while the AC-4b pattern and non-AC-4 and AC-0 patterns were observed in 11 (18.9%), 30 (51.7%), and 1 (1.7%) of these samples, respectively. It is reasonable to presume that the presence of extra autoantibodies may have contributed to the low frequency of the AC-4a pattern in this subset of anti-SS-A/Ro-positive samples.

### Brazilian Branch

**Figure 7:**
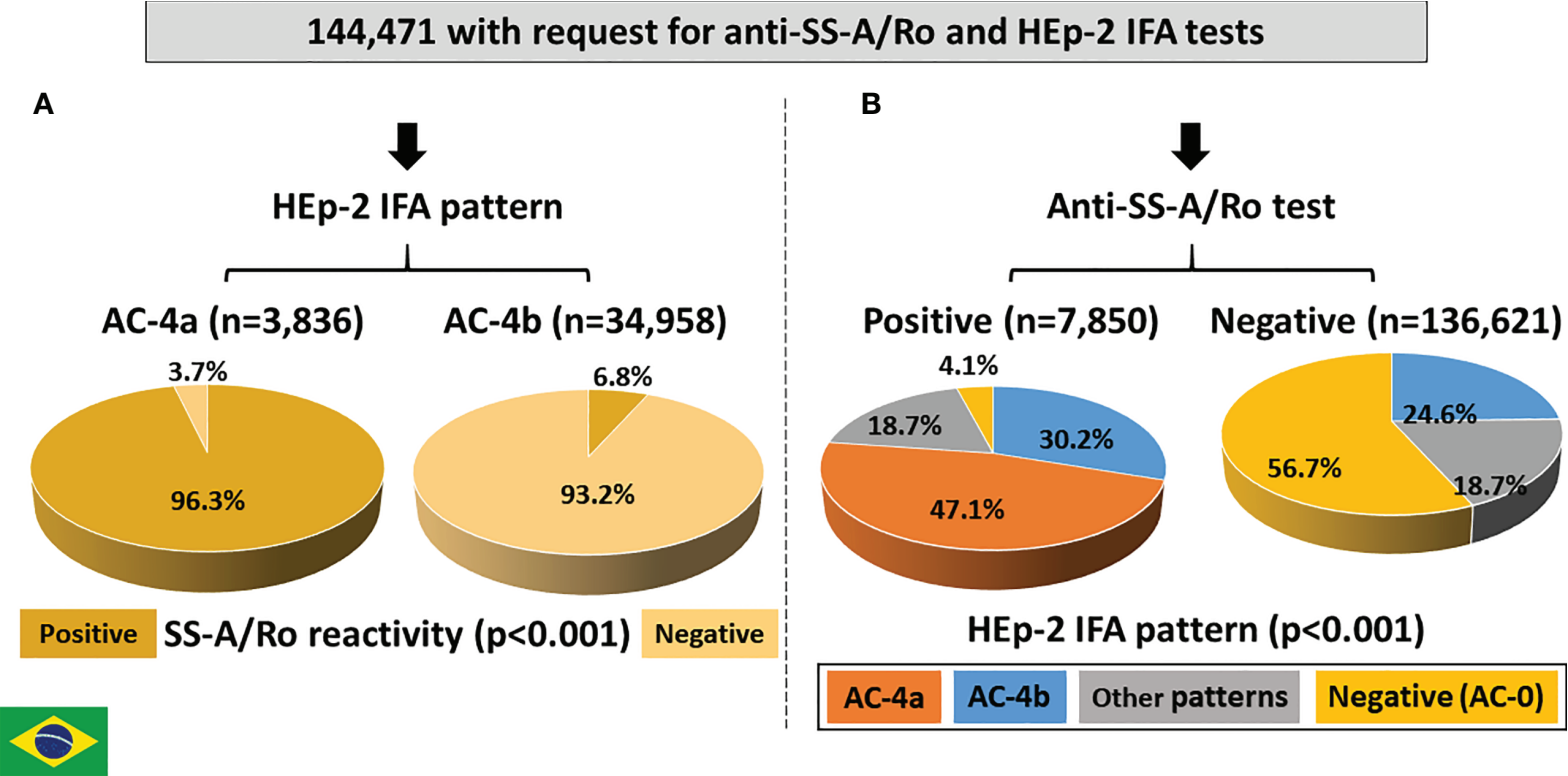
Association between the AC4a pattern and antibodies to Ro60 according to data from Fleury Laboratory, São Paulo, Brazil. **(A)** HEp-2 IFA pattern perspective: most samples with the AC-4a pattern were positive, whereas most samples with the AC-4b were negative for anti-Ro60. **(B)** Anti-Ro60 antibody perspective: the AC-4a pattern is the most frequent pattern in samples positive for anti-Ro60 and was observed in only 0.1% of samples with no reactivity to Ro60.

From January 2012 to July 2018, 144,471 records had concomitant request of the HEp-2 IFA and anti-SS-A/Ro tests. The AC-4a pattern was much less frequent (3,836; 2.7%) than the AC-4b pattern (34,958; 24.2%) (**Figure 7A**). Although less frequent, the AC-4a pattern was very specific for anti-SS-A/Ro antibodies, as reactivity to SS-A/Ro was observed in 3,692 (96.3%) of the AC-4a samples as opposed to only 2,363 (6.8%) of those with the AC-4b pattern.

Among the 7,850 samples with positive reactivity to SS-A/Ro, 3,694 (47.1%) presented the AC-4a pattern, 2,371 (30.2%) presented the AC-4b pattern, 1,467 (18.7%) presented other patterns and 318 (4.1%) presented a negative HEp-2 IFA result (**Figure 7B**). In contrast, among the 136,621 samples with a negative result for anti-SS-A/Ro, 140 (0.1%) presented the AC-4a pattern, 33,541 (24.6%) presented the AC-4b pattern, 25,431 (18.6%) presented other patterns, and 77,509 (56.7%) presented a negative HEp-2 IFA result. The difference in HEp-2 IFA pattern distribution between samples with and without reactivity to SS-A/Ro was statistically significant.

**Figure 8:**
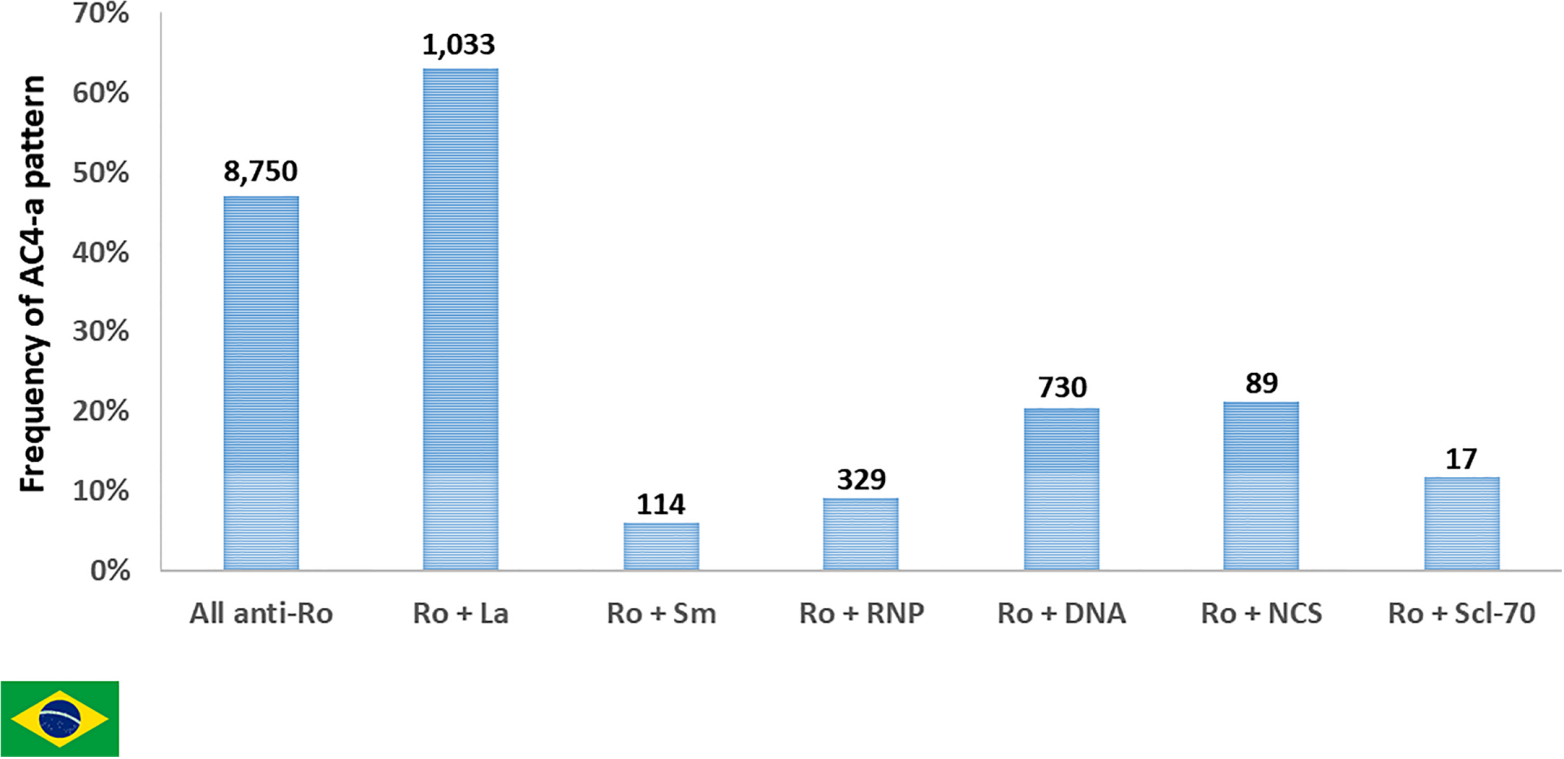
Frequency of pattern AC-4a in samples reactive with Ro60 and other autoantibodies according to data from Fleury Laboratory, São Paulo, Brazil. A nested cohort of 2,312 Ro60-positive samples had antibodies to one additional DAA (SS-B/La, dsDNA, nucleosome/chromatin, Sm, U1-RNP, or Scl-70). The frequency of samples presenting the AC-4a pattern is depicted for the combinations of SS-A/Ro and each autoantibody. Absolute numbers in each category are shown at the top of columns.

The presence of additional autoantibodies in the sample may modulate the final HEp-2 IFA pattern. This may cause several anti-SS-A/Ro-reactive samples to produce HEp-2 IFA patterns other than AC-4a. In fact, some of the 7,850 anti-SS-A/Ro-positive samples had information of concomitant presence of antibodies to native DNA, nucleosome, Sm, U1-RNP or Scl-70. As expected, the frequency of the AC-4a pattern decreased in such samples in comparison to all samples with anti-SS-A/Ro reactivity. In contrast, the concomitant presence of anti-SS-B/La antibodies did not affect the frequency of the AC-4a pattern (**Figure 8**).

We further explored this point, by analyzing a nested cohort of 2,953 samples with coexistent results for HEp-2 IFA and antibodies to all of the following DAA (native DNA, nucleosome, Sm, U1-RNP, Ro60, SS-B/La, and Scl-70). Except for minor details, this nested cohort analysis reproduced the above analysis with all cases tested for anti-Ro60 antibodies. As demonstrated in **Table 1**, the frequency of Ro60 antibodies is much higher in samples with the AC-4a pattern (97.2%) than in samples with the AC-4b pattern (9.7%). Although highly specific for Ro60 antibodies, the AC-4a pattern was not very sensitive, as it occurred in only 70 (29.3%) of 188 Ro60-reactive samples with no other DAA. In fact, the majority of the “monospecific” Ro60-reactive samples in this nested cohort presented assorted patterns (52.7%), while a minor fraction presented the AC-4b pattern (14.4%) or the AC-1 pattern (11.5%). Curiously, four “monospecific” Ro60-reactive samples presented a negative (AC-0) HEp-2 IFA test, but it should be noted that AC-0 was also observed in samples reactive with nucleosome (*n* = 14), native DNA (*n* = 3), and Sm/RNP (*n* = 1). Of note, only two samples with the AC-4a pattern showed no DAA, as opposed to the AC-4b pattern, with 86.2% of the samples presenting no DAA (**Table 1**).

**Table 1:** Distribution of HEp-2 IFA patterns in 2,953 samples with order for disease-associated autoantibodies (DAA)* in the Brazilian series.

DAA present	HEp-2 IFA patterns					*p*-value**
	AC-4a	AC-4b	AC-1	Other	AC-0	
All Ro60	70 (97.2)^§^	38 (9.7)	34 (15.8)	148 (11.9)	5 (0.5)	<0.001
Ro60 + SS-B/La	7 (10)	4 (7.4)	2 (1.3)	12 (1.0)	0	NS
Ro60 alone	55 (76.4)	27 (6.9)	3 (1.4)	99 (7.9)	4 (0.4)	<0.001
RNP/Sm	0	4 (1.0)	10 (4.7)	56 (4.5)	1 (0.1)	NS
Native DNA	1 (1.4)	10 (2.6)	64 (29.8)	43 (3.4)	3 (0.3)	<0.001
Nucleosome	7 (9.7)	17 (4.3)	144 (67)	99 (7.9)	14 (1.4)	<0.001
Scl-70	0	0	0	2 (0.2)	0	NS
No DAA	2 (2.8)	338 (86.2)	64 (29.8)	980 (78.6)	1,006 (98)	<0.001
Total	72 (100)	392 (100)	215 (100)	1,247 (100)	1,027 (100)	

*All samples were tested for the following disease-associated autoantibodies (DAA): Ro60, SS-B/La, Sm, U1-RNP, Scl-70, native DNA, and nucleosome. **The statistics compared the frequency of patterns in samples with a given autoantibody vs. all the other samples negative for that autoantibody. ^§^Percentages represent the frequency of any given autoantibody specificity in relation to the total of samples with the respective pattern. NS, non-significant.

It is interesting to compare these results with the analysis of patterns traditionally accepted to exhibit strong association with autoantibody specificities, such as the case of AC-1, which is accepted to present a strong association with antibodies to native DNA and nucleosome. As seen in **Table 1**, the correspondence is not absolute. Although the majority of samples presenting the AC-1 pattern have reactivity to native DNA (29.8%) and/or to nucleosome (67%), 29.8% of them presented none of the DAA. In addition, only 52.9% and 51.2% of samples with reactivity to native DNA and nucleosome, respectively, presented the AC-1 pattern. Almost half of the samples reacting to native DNA and/or nucleosome presented assorted patterns and a few of them presented no HEp-2 IFA reactivity (AC-0).

### German Branch

**Figure 9:**
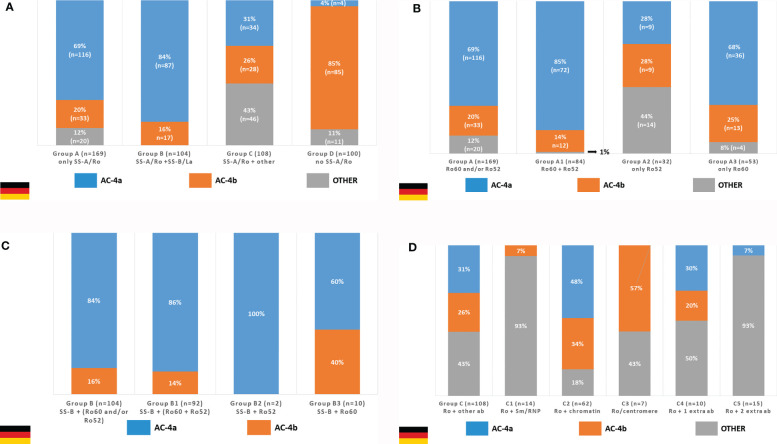
Frequency of the AC4a pattern and other patterns in samples with different combination of autoantibodies according to data from the Technical University Dresden, Germany. **(A)** The AC-4a pattern is more frequent in samples with anti-SS-A/Ro only or in combination with anti-SS-B/La as compared with samples with anti-SS-A/Ro plus other autoantibodies (dsDNA, nucleosome, Sm, U1-RNP, SS-B/La, Scl-70, Jo-1) and samples with no reactivity to SS-A/Ro. **(B)** In the group with sole reactivity to SS-A/Ro, those that contain anti-Ro60 antibodies show higher frequency of the AC-4a pattern than those with exclusive reactivity to Ro52. **(C)** The coexistence of anti-SS-B/La did not disturb the association of anti-SS-A/Ro antibodies and the AC-4a pattern. **(D)** The presence of other DAA prevented the appearance of the AC-4a pattern in the majority of SS-A/Ro-reactive samples.

Out of 742 SS-A/Ro-positive samples with result for HEp-2 IFA, 381 were available for further testing. The AC-4a pattern was very frequent in samples with exclusive presence of anti-SS-A/Ro antibodies and in those with concurrent antibodies to SS-A/Ro and SS-B/La, but it dropped to 1/3 of the samples with anti-SS-A/Ro plus additional non-SS-B/La autoantibodies (**Figure 9A**). In contrast, the AC-4a pattern was seldom observed in samples with no anti-SS-A/Ro reactivity, supporting the specificity of the AC-4a pattern to anti-SS-A/Ro antibodies (**Figure 9A**, group D). Among the 100 samples from this group, the AC-4a pattern occurred in only four samples (4%), two of them with additionally stained mitotic chromatin plate; the AC-4b pattern occurred in 85 samples (85%), 11 of them with additionally stained chromatin plate; and other patterns occurred in 11 samples (11%).

Among the 169 samples in group A (monospecific anti-SS-A/Ro), the AC-4a pattern occurred in 116 cases (68.6%), the AC-4b pattern occurred in 33 cases (19.5%), and other patterns (including AC-0) occurred in 20 cases (11.8%). The frequency of the AC-4a pattern was even greater among the 84 samples presenting reactivity to Ro60 and Ro52 (subgroup A1), where the AC-4a pattern occurred in 71 cases (84.5%). AC-4a was more frequent in the 53 samples monospecific for the Ro60 (subgroup A3), where it occurred in 36 cases (67.9%), than in the 32 samples reacting only with the Ro52 (subgroup A2), where the AC-4a pattern occurred in 9 cases (28.1%) (**Figure 9B**).

The association of the AC-4a pattern with antibodies to SS-A/Ro was not disturbed by the presence of anti-SS-B/La antibodies. In fact, among the 104 samples reactive to SS-A/Ro and SS-B/La (group B), the AC-4a pattern was observed in 87 samples (83.7%) and the AC-4b pattern was observed in 17 samples (16.3%). Within this group, the strong association with the AC-4a pattern was seen irrespective of the combination of reactivity to the Ro60 and Ro52 (**Figure 9C**). However, it must be noted that subgroup B2 (reactive with SS-B/La and Ro52) comprised only two samples.

On the other hand, the concurrent presence of non-SS-B/La autoantibodies tended to disturb the display of the AC-4a pattern in SS-A/Ro-positive samples. Indeed, among the 108 sera reactive to SS-A/Ro and at least one more non-SS-B/La autoantibody (group C), the AC-4a pattern was observed in 34 samples (31.5%), whereas the AC-4b pattern was seen with 28 samples (25.9%), and other patterns in 46 samples (42.6%) (**Figure 9D**, first column). Samples with a combination of anti-SS-A/Ro and anti-chromatin antibodies (subgroup C2) showed the highest frequency of AC-4a pattern (48%). It must be emphasized, however, that in this group, the mitotic chromosome plate was stained in a homogeneous fashion in the majority of samples showing the AC-4a (91.2%) and AC-4b patterns (89.3%). Among the 10 samples in subgroup C4 (presenting one concurrent non-SS-B/La antibody), the AC-4a pattern could be recognized in three samples: one with anti-Jo-1 antibodies, one with anti-mitotic spindle reactivity, and one with anti-dsDNA antibodies. The AC-4a pattern could not be identified in the remaining samples of this group with reactivity to Jo-1 (*n* = 2), mitotic spindle (*n* = 1), centriole (*n* = 1), Scl-70 (*n* = 1), Ku (*n* = 1), and nuclear envelope (*n* = 1), respectively. There were 15 SS-A/Ro-positive samples with concurrent presence of more than one non-SS-B/La antibody (subgroup C5), among which only one (reacting to SS-A/Ro, dsDNA, histone, Scl-70, Sm, and Jo1) presented the AC-4a pattern.

Altogether, the data from the three clinical centers converged in showing that samples with the AC-4a pattern have a higher frequency of anti-SS-A/Ro antibodies than those with the AC-4b pattern. Conversely, the AC-4a pattern was more frequent and the AC-4b pattern was less frequent in samples with anti-SS-A/Ro antibodies in comparison to those lacking this autoantibody specificity (**Table 2**). It should be noticed that the frequencies observed in the three centers are not formally comparable, because of heterogeneity in the methodological strategy causing differences in some characteristics of patients in the three centers. For example, 79.2% of the samples in the German center were positive for anti-SS-A/Ro antibodies, as opposed to 25.1% and 5.4% in the Argentinian and Brazilian centers, respectively. This was expected because a positive reactivity to SS-A/Ro was a leading criterion in the selection of the German samples.

**Table 2:** Summary of data on the association of AC-4a and AC-4b patterns with reactivity to SS-A/Ro in the three clinical centers*.

	Durand Hospital	Fleury Laboratory	TU Dresden
Study design	Databank analysis	Databank analysis	Sample analysis
Number of samples/records	383	144,471	481
**AC patterns in samples with reactivity to SS-A/Ro**			
Frequency of α-SS-A/Ro 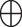 (% over total samples)	96 (25.1%)	7,850 (5.4%)	381 (79.2%)
Frequency of AC-4a pattern	23 (24%)	3,694 (47.1%)	237 (62.2%)
Frequency of AC-4b pattern	23 (24%)	2,371 (30.2%)	78 (20.5%)
Frequency of other patterns	50 (52.1%)	1,785 (22.7%)	66 (17.3%)
**AC patterns in samples not reacting to SS-A/Ro**			
Frequency of α-SS-A/Ro Ø (% over total samples)	287 (74.9%)	136,621 (94.6%)	100 (20.8%)
Frequency of AC-4a pattern	11 (3.8%)	140 (0.1%)	4 (4%)
Frequency of AC-4b pattern	77 (26.8%)	33,541 (24.6%)	85 (85%)
Frequency of other patterns	199 (69.3%)	102,940 (75.3%)	11 (11%)
**Frequency of anti-SS-A/Ro reactivity in samples with the AC4-a pattern**			
Frequency of AC-4a pattern (% over total samples)	34 (8.9%)	3,836 (2.7%)	241 (63.3%)
Frequency of anti-SS-A/Ro 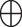	23 (67.6%)	3,692 (96.3%)	237 (98.3%)
**Frequency of anti-SS-A/Ro reactivity in samples with the AC4-b pattern**			
Frequency of AC-4b pattern (% over total samples)	100 (26.1%)	34,958 (24.2%)	163 (42.8%)
Frequency of anti-SS-A/Ro 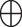	23 (23%)	2,363 (6.8%)	78 (47.9%)

*This table provides an overview of the results from the three centers and should not be used for a formal comparison, because the selection criteria are different for the three centers.

## Discussion

ICAP has achieved considerable progress in the last 6 years by promoting the harmonization of the nomenclature of 30 HEp-2 IFA patterns that have been organized into a structured algorithm with hyperlink to information related to the description of the patterns, representative images, their associations to distinctive autoantibodies, and their clinical relevance (6, 7). Along the sequential ICAP workshop meetings, the classification algorithm has been improved and new patterns have been incorporated, such as the AC-0 (negative result) (26) and the AC-29 (topoisomerase I-like pattern) (11). The present international multicenter study provides evidence that there is an opportunity for further improving the classification of the fine speckled nuclear pattern (AC-4) by showing that the myriad discrete fine speckled nuclear pattern (preliminarily designated AC-4a) is associated with anti-Ro60 antibodies whereas the plain fine speckled nuclear pattern (preliminarily designated AC-4b) is not.

Dellavance et al. originally reported on a variant of the fine speckled nuclear pattern characterized by myriad tiny discrete nuclear speckles (15). They reported a strong association of this pattern with the presence of anti-SS-A/Ro antibodies and accordingly designated it as SS-A/Ro-like pattern. The study was appropriately controlled, the observations were done by blinded experts, and the findings were consistent in that the association was confirmed in a bidirectional manner, i.e., starting from samples with a positive result for anti-SS-A/Ro antibodies or from samples yielding a SS-A/Ro-like pattern in HEp-2 IFA. Nonetheless, the data were obtained in a single center, which may not reflect the experience of analysts in other institutions. The present study addresses this limitation by incorporating the data from three independent international expert clinical laboratories and experimental evidence from a fourth laboratory. The clinical laboratory data were derived from the databank referent to several years of the routine operation of the three laboratories, which tends to reflect an unbiased real-life scenario.

Although the methodological approach taken by the three clinical laboratories is different, the results point to several common points that support the legitimacy in the acknowledgment of the AC-4a pattern. The three centers uniformly observed that the AC-4a pattern, but not the AC-4b pattern, was more frequent in samples with than in those without anti-SS-A/Ro antibodies. Conversely, samples presenting SS-A/Ro antibodies most frequently yielded the AC-4a pattern, whereas samples negative for anti-SS-A/Ro frequently yielded the AC-4b pattern and seldom the AC-4a pattern. Data from the three centers showed that the coexistence of antibodies to DAA (dsDNA, chromatin, histones, Sm, U1-RNP, Scl-70, and CENP-B) tended to conceal the characteristics of AC-4a. On the other hand, the coexistence of antibodies to SS-B/La did not disturb the association of anti-SS-A/Ro antibodies with the AC-4a pattern. In fact, data from the three clinical laboratory centers showed higher frequency of the AC-4a pattern in samples that had anti-SS/A-Ro and anti-SS-B/La antibodies than in those with anti-SS-A/Ro only. Of note, the six monospecific anti-SS-B/La-positive samples from the Argentinian center did not yield the AC-4a pattern. Considering the usually conjugated response to SS-A/Ro in SS-B/La-positive patients, we hypothesize that the presence of anti-SS-B/La antibodies signalizes a more robust response to SS-A/Ro, favoring a higher frequency of the AC-4a pattern in samples with both antibodies in comparison to those with anti-SS-A/Ro only, as observed in the three centers.

The Argentinian and German centers explored the fine specificity of SS-A/Ro by analyzing samples according to the reactivity to Ro60 and Ro52. Samples reacting to Ro60 confirmed the strong association with the AC-4a pattern. To our surprise, some samples that reacted only to Ro52 also showed a relevant association with the AC-4a pattern, although a substantial fraction of these samples showed the AC-4b, other patterns or no reactivity. This is intriguing because Ro52 has no nuclear localization signal and full-length Ro52-transfected cells showed Ro52 predominantly in the cytoplasm (27). Using three anti-Ro52 monoclonal antibodies, Schmitz et al. obtained a predominantly cytoplasmic diffuse speckled staining in human bladder epithelial cell lines RT112 as well as in HEp-2 cells, and predominantly multiple discrete nuclear pattern on human cell line XPTA (28). In fact, the literature indicates that human antibodies to Ro60 consistently yield a positive nuclear staining, but there is controversy regarding the HEp-2 IFA reactivity of antibodies against Ro52. Some studies report a positive cytoplasmic, nucleolar, or nuclear HEp-2 staining in samples positive for anti-Ro52 and negative for anti-Ro60; however, these samples were not systematically investigated to exclude the presence of other relevant autoantibodies (29–31). Human affinity-purified anti-Ro52 antibodies from five patients yielded no relevant staining while anti-Ro52 from two patients showed predominantly nuclear staining on XPTA cells (28). In addition, it should be emphasized that solid-phase immunoassays frequently do not detect autoantibodies that are detected by HEp-2 IFA and immunoprecipitation. Therefore, it is likely that the samples classified as monospecific anti-Ro52 in the present study actually contained anti-Ro60 antibodies that reacted in HEp-2 IFA, yielding the expected AC-4a pattern. In fact, Chan and Buyon showed that so-called “monospecific” anti-Ro52 samples from patients with SjS and/or SLE depict anti-Ro60 reactivity and co-precipitation of the Ro60 associated hY-RNAs upon careful analysis in immunoprecipitation (32, 33). Of interest, Dellavance et al. found that none of 13 samples from patients with autoimmune hepatitis with exclusive reactivity to Ro52 had a relevant reactivity in HEp-2 IFA (15). In the scenario of autoimmune hepatitis, it is likely that these samples indeed did not contain anti-Ro60 antibodies or other autoantibodies to HEp-2 antigens.

Although the herein presented results confirm the report from Dellavance et al. (15), it is clear that the association between the AC-4a pattern and anti-SS-A/Ro antibodies is not absolute. In fact, this is in accordance with similar findings for other HEp-2 IFA patterns with well-defined and widely accepted autoantibody associations, emphasizing the fact that the phenomenon of HEp-2 IFA pattern/autoantibody association is not absolute. Unexpected HEp-2 IFA patterns in samples with a given autoantibody specificity are not rare, as observed in the daily clinical laboratory routine by experts. Recently, Prado et al. examined this aspect with regard to the AC-1 pattern and showed that only 94 of 194 (48.5%) SLE samples with antibodies to dsDNA and/or nucleosome showed the AC-1 pattern in the HEp-2 IFA test (34). By exploring further the basis of the association, they found that samples with high titer antibodies to both dsDNA and nucleosomes had the highest probability of presenting the AC-1 pattern. Therefore, taking into consideration the relative immunologic associations of other HEp-2 IFA patterns, we consider that the association rate of the AC-4a pattern with anti-SS-A/Ro antibodies herein reported is within the expected range observed for other HEp-2 IFA patterns considered clinically relevant.

The novel AC-4a pattern has the advantage of discriminating the immunological and clinical relevance of two closely related HEp-2 IFA patterns that appear as a fine speckled nuclear pattern with no staining of the metaphase chromatin mass. The AC-4a myriad discrete fine speckled nuclear pattern, but not the AC-4b plain fine speckled nuclear pattern, is associated with anti-SS-A/Ro antibodies. As with other HEp-2 IFA patterns, the correct identification of the AC-4a pattern can be helpful in indicating the possible clinical relevance and the reflex autoantibody testing to be performed. The detailed knowledge about special patterns, such as the AC-4a variant, will help to create and improve algorithms for automated pattern recognition systems or computer-aided diagnosis systems, which may support the human observer and facilitate objectivity. The first steps in this direction are the precise description of patterns and their relation to specific autoantibody entities and the potential influence of further autoantibodies, assay brands, and other factors on the recognition of a pattern.

One potential limitation of the study is the fact that the methodology is heterogeneous among the participating centers. However, we understand that this is also one of the strengths of the study as it reflects the real-world experience in which there is wide variability in operator expertise, microscopes and HEp-2 kits used, among other factors. The three centers used different HEp-2 IFA kits, different methods for the determination of anti-SS-A/Ro (including discrimination or not of anti-Ro52 and anti-Ro60), and other DAA. This also applies to the selection of samples, as the German center processed samples retrieved from the serum bank whereas the Argentinian and Brazilian centers retrospectively analyzed the databank from samples processed in the routine operation. The observed association of the AC-4a pattern and reactivity to SS-A/Ro in the three centers, despite differences in the methodology, speaks for the generalization of the validation of this association.

The heterogeneity in the methodology possibly affected the strength of the association between the AC-4a pattern and reactivity to SS-A/Ro observed in the three centers. In particular, heterogeneity in observer expertise probably accounted for the variability of the results, as the Brazilian center reported the AC-4a pattern in 2013 (15) and regularly classifies this pattern in the routine operation since then. As observed with other HEp-2 IFA patterns, the recognition of the AC-4a pattern requires apprenticeship and training. This is especially true in this case, because the distinction between the AC-4a and AC-4b patterns is a subtle difference in the texture of the nuclear staining pattern. Of relevance to this study, in our day-to-day practice at the Brazilian and Argentinian centers, the analysts are recommended to ascribe the AC-4a pattern only when the typical features are observed; otherwise, the samples should be classified as AC-4b. In this study, the German center found that seven samples could not be clearly classified in the first assay and needed to be reprocessed and further titrated, eventually allowing the final classification of the pattern. In our experience, the characteristics of AC-4a may not be evident at the screening 1/80 dilution and become clearer as the sample is further diluted. In general, the 400 times magnification is appropriate for identification of the AC-4a pattern. In addition, we noticed that AC-4a is more evident with certain HEp-2 slide brands than with others, and this may depend on distinct details of the cell culture and fixation methods applied by different manufacturers (35). Therefore, one needs to identify how the AC-4a pattern shows up in the particular HEp-2 slide brand in use in the laboratory. The availability of the IUIS/ASC reference serum IS2105 will also help laboratories to identify this AC-4a pattern efficiently.

In conclusion, this multicenter study confirms the previously reported strong association of the myriad discrete fine speckled nuclear pattern and antibodies to SS-A/Ro in opposition to the plain fine speckled nuclear pattern, which appears to have no circumscribed autoantibody association. The similarity of results in three independent international expert clinical laboratories speaks for the worldwide applicability of these two variants of the AC-4 pattern. We propose that these novel patterns are incorporated into the ICAP classification algorithm with the codes AC-4a and AC-4b, respectively. The AC-4 pattern should be maintained as an umbrella pattern for cases in which one cannot safely discriminate between AC-4a and AC-4b patterns. The acknowledgment of the AC-4a pattern should add value to the interpretation of the HEp-2 IFA test.

## Data Availability Statement

The datasets presented in this article are not readily available as part of the data refer to the databank from private laboratories that do not allow sharing the data. Requests to access the datasets should be directed to LA (luis.andrade@unifesp.br).

## Ethics Statement

The studies involving human participants were reviewed and approved by Ethics Committee of the Medical Faculty Carl Gustav Carus of the TU Dresden (EK 56022014 and EK 226112006) and of the Sächsische Landesärztekammer (EK-BR-13/13-1), as well as the Ethics Committee at Durand Hospital and Fleury Laboratory. Written informed consent for participation was not required for this study in accordance with the national legislation and the institutional requirements.

## Author Contributions

Study design: LA, NR, OC, and EC. Data acquisition: LA, NR, AD, FI, OC, M-LR, and EC. Manuscript writing: LA, NR, and EC. Manuscript review: LA, NR, AD, FI, OC, M-LR, EC, and KC. All authors contributed to the article and approved the submitted version.

## Funding

LA receives grant PQ-1D 310334/2019-5 from the Brazilian National funding agency CNPq.

## Conflict of Interest

The authors declare that the research was conducted in the absence of any commercial or financial relationships that could be construed as a potential conflict of interest.

## Publisher’s Note

All claims expressed in this article are solely those of the authors and do not necessarily represent those of their affiliated organizations, or those of the publisher, the editors and the reviewers. Any product that may be evaluated in this article, or claim that may be made by its manufacturer, is not guaranteed or endorsed by the publisher.
